# Active Calcium/Calmodulin-Dependent Protein Kinase II (CaMKII) Regulates NMDA Receptor Mediated Postischemic Long-Term Potentiation (i-LTP) by Promoting the Interaction between CaMKII and NMDA Receptors in Ischemia

**DOI:** 10.1155/2014/827161

**Published:** 2014-03-10

**Authors:** Ning Wang, Linlin Chen, Nan Cheng, Jingyun Zhang, Tian Tian, Wei Lu

**Affiliations:** ^1^Department of Neurobiology, Nanjing Medical University, Nanjing, China; ^2^Key Laboratory of Developmental Genes and Human Disease, Institute of Life Sciences, Southeast University, Nanjing, China

## Abstract

Active calcium/calmodulin-dependent protein kinase II (CaMKII) has been reported to take a critical role in the induction of long-term potentiation (LTP). Changes in CaMKII activity were detected in various ischemia models. It is tempting to know whether and how CaMKII takes a role in NMDA receptor (NMDAR)-mediated postischemic long-term potentiation (NMDA i-LTP). Here, we monitored changes in NMDAR-mediated field excitatory postsynaptic potentials (NMDA fEPSPs) at different time points following ischemia onset *in vitro* oxygen and glucose deprivation (OGD) ischemia model. We found that 10 min OGD treatment induced significant i-LTP in NMDA fEPSPs, whereas shorter (3 min) or longer (25 min) OGD treatment failed to induce prominent NMDA i-LTP. CaMKII activity or CaMKII autophosphorylation displays a similar bifurcated trend at different time points following onset of ischemia both *in vitro* OGD or *in vivo* photothrombotic lesion (PT) models, suggesting a correlation of increased CaMKII activity or CaMKII autophosphorylation with NMDA i-LTP. Disturbing the association between CaMKII and GluN2B subunit of NMDARs with short cell-permeable peptides Tat-GluN2B reversed NMDA i-LTP induced by OGD treatment. The results provide support to a notion that increased interaction between NMDAR and CaMKII following ischemia-induced increased CaMKII activity and autophosphorylation is essential for induction of NMDA i-LTP.

## 1. Introduction

Ischemic stroke, a brain attack induced by the reduction of blood flow, is one of the leading causes of death and disability worldwide [1]. Unfortunately, the mechanisms underlying stroke processing are less understood, and there are no effective treatments targeting it. Following stroke, a pathological neural plasticity termed postischemic long-term potentiation (i-LTP) often occurs over time [2, 3]. And emerging evidences from animal models suggest that such i-LTP plays important roles in both injury and recovery. Thus, it is necessary to improve the comprehension of the mechanisms mediating i-LTP after stroke.

It is generally accepted that the pathological plasticity initiated by excessive calcium influx follows the activation of NMDAR after stroke [4]. Over the past two decades, though there are many published papers reporting the phenomenon termed NMDAR-mediated i-LTP, most researchers focused on the detailed mechanisms and significant implication in NMDAR dependent postischemic plasticity while NMDAR-mediated response got less attention [5, 6]. In previous articles, NMDAR fEPSPs were isolated in low-magnesium ACSF perfusion medium in presence of GABA_A_R antagonist BMI (10 *μ*M) and AMPAR antagonist NBQX (10 *μ*M). NMDAR antagonist D-APV was selected to identify whether NMDAR mediated i-LTP by recording this in ACSF perfusion medium [7, 8]. It has been favored that there was cascade response followed by calcium influx in i-LTP [9]; the conspicuous response is CaMKII activation and autophosphorylation which is involved in i-LTP after ischemia [9, 10].

In addition, it has been reported that the component of postsynaptic NMDARs changes in i-LTP and some researchers have proved the GluN2B-containing NMDAR plays an important role in i-LTP [11, 12]. The specific GluN2B antagonist ifenprodil exhibits a dose dependent inhibition of i-LTP, as well as the lower infarction volume. In the early studies, CaMKII activation and autophosphorylation initiated the succedent association between NMDAR and CaMKII and the sites of their interaction have been reported clearly in activity-dependent forms of synaptic plasticity, long-term potentiation (LTP), and long-term depression (LTD), proposed as the mechanism of learning and memory [13–15]. CaMKII has been widely studied as GluN2B binding protein in promoting the translocation of GluN2B to postsynaptic sites [16–18]. And it was clarified that both NMDAR antagonist and CaMKII inhibitor reduced the targeting of CaMKII to GluN2B, as well as the NMDAR translocation to postsynaptic membrane [11]. In summary, we emphasized on the most important question that whether this mechanism is involved in pathological neural plasticity or not.

On the other hand, the time points play an important role in ischemia injury and recovery. However, it still fails to form a consensus that multiple time points bring about different NMDAR-mediated i-LTP. After the induction, the mechanisms of the NMDAR-mediated i-LTP are not clear. The ability of recovery from ischemia is proposed to depend on the duration of the injury [19]. OGD treatment for 9 min and 14 min exhibited different phenomena. The shorter the OGD lasted, the easier the function could recover. Otherwise, after occlusion for 90 sec and 90 min, there were distinctions between the levels of *α*CaMKII phosphorylation at T286 site. The phosphorylation of Thr286-*α*CaMKII was significantly improved by 90 sec occlusion, while the phosphorylation of Thr286-*α*CaMKII was apparently decreased by 90 min occlusion [20].

In this study, the NMDAR-mediated i-LTP induced by OGD was demonstrated by employing electrophysiological recordings in hippocampal slices. An apparent increase of NMDAR in postsynaptic membrane caused by photothrombotic lesion (PT) was observed through Western blotting. The activity and autophosphorylation levels of CaMKII at different time points after ischemia were determined on OGD and PT models. Furthermore, using short disturbing peptides, it was found that the active CaMKII promotes the interaction between CaMKII and NMDARs and regulates NMDAR-mediated i-LTP in ischemia. Our data deepens the comprehension of the pathological plasticity after cerebral ischemia and provides useful experimental results for stroke therapeutics.

## 2. Materials and Methods

### 2.1. Hippocampal Slice

All animals and experimental protocols were carried out by the guidance of the National Institutes of Health (NIH) for the Care and Use of Laboratory Animals. Adult C57BL/6 mice were anesthetized with 1% pelltobarbitalum natricum and decapitated. The entire brain was quickly removed. Brain slices (350 *μ*m thickness) were cut on a vibratome (VT1000S, Leica, Germany) in ice-cold artificial cerebrospinal fluid (ACSF) containing (in mM) 126 NaCl, 2.5 KCl, 1 MgCl_2_, 1 CaCl_2_, 1.25 KH_2_PO_4_, 26 NaHCO_3_, and 20 glucose, pH 7.4, which was gassed with 95% O_2_ and 5% CO_2_. The fresh slices were incubated in a chamber with oxygenated ACSF and were recovered at 34°C. The slices were stored homogenized in cold 0.32 M sucrose containing 1 mM HEPES, 1 mM MgCl_2_, 1 mM NaHCO_3_, 20 mM sodium pyrophosphate, 20 mM *β*-phosphoglycerol, 0.2 mM dithiothreitol, 1 mM EDTA, 1 mM EGTA, 50 mM NaF, 1 mM Na_3_VO_4_, and 1 mM p-nitrophenyl phosphate, pH 7.4, in the presence of the following protease inhibitors and phosphatase inhibitors: 1 mM phenylmethylsulfonyl fluoride (PMSF), 5 g/mL aprotinin, 5 g/mL leupeptin, 5 g/mL pepstatin A, and 16 g/mL benzamidine. The homogenate was centrifuged at 1,000 ×g for 10 min and the supernatant was collected. The content of protein was measured using a BCA protein assay (Pierce, USA) [21].

### 2.2. Western Blotting and Coimmunoprecipitation

Slices used for coimmunoprecipitation analyses were preincubated with Tat-GluN2B (ChinaPeptides). Total protein was incubated with antibody against CaMKII (2 *μ*g) overnight at 4°C in immunoprecipitation buffer (0.05 M HEPES, pH 7.4, containing 10% glycerol, 0.15 M NaCl, 1% TritonX-100, 0.5% Nonidet P-40, and 1 mM concentration of each of EDTA, EGTA, PMSF, and Na_3_VO_4_). Then protein A/G-agarose beads were added, mildly vortexed, and incubated for 2 h at 4°C. The beads were recovered by centrifugation at 12,000 ×g and gently washed three times with immunoprecipitation buffer. The left beads were treated the same as Western blot. For Western blot, total protein (20 *μ*g) was boiled and was separated with 7.5% SDS-PAGE and transferred onto a PVDF membrane. Membranes were blocked in 3% (w/v) BSA (fraction V) in TBST (0.1% Tween 20) for 1 hr at room temperature (RT). Detection antibodies for Western blot analysis were from Biotime (*β*-Tubulin, 1 : 3000), Santa Cruz (p-CaMKII, 1 : 800), and Santa Cruz (GluN1, 1 : 800). Conjugated antibodies were from GE Healthcare (anti-mouse IgG-HRP, 1 : 4000) and Santa Cruz (rabbit anti-goat IgG-HRP, 1 : 8000). Signals were visualized with ECL kit (Amersham Biosciences). Band intensities were quantified by ImageJ (NIH, USA).

### 2.3. Electrophysiological Recordings in Acute Slices

Field excitatory postsynaptic potentials (fEPSPs) recordings were made as previous researches described [22]. Briefly, a stimulating electrode was located in Schaffer collateral; a recording pipette filled with 3 mM NaCl was put in stratum radiatum of CA1. Bicuculline methiodide (BMI, 10 *μ*M) was from Tocris; 1,2,3,4-tetrahydro-6-nitro-2,3-dioxobenzo[*f*]-quinoxaline-7-sulfonamide (NBQX, 10 *μ*M) was from Sigma; Tat-GluN2B was from ChinaPeptides.

### 2.4. Oxygen-Glucose-Deprivation-Induced Ischemia Model

The slices were deprived of anoxia/hypoglycemia by replacing 95% O_2_/5% CO_2_ with 95% N_2_/5% CO_2_ and switching to an ACSF solution containing 20 mM sucrose instead of glucose for 5 to 10 minutes.

### 2.5. Photothrombotic Lesion

The female C57BL/6 mice (8–12 weeks) weighing 20–25 g were anesthetized using 1% pelltobarbitalum natricum with the body dose of 50 mg/kg. The scalps of anesthetized animals were exposed through shaving the mice hair. Before illumination, 1% Rose Bengal (Sigma) dissolved in 0.9% saline was intraperitoneally infused via the body dose of 100 mg/kg. Subsequently, focal illumination of the brain continued for 15 minutes with a strong cold light source through the intact skull leading to focal infarcts ranging from cortical to hippocampal area.

### 2.6. TTC Staining

The infarct size, location, and geometry of mice were measured 24 h after photothrombotic lesion. After anesthetization and execution, brains were removed directly and frozen at −20°C for 5 min. The whole brains of mice were incised to corresponding slices at 2 mm and sections were immersed in 1% 2,3,5-triphenyltetrazolium chloride (TTC; Sigma) at 37°C for 20 min in light-blocking environment. The presence or absence of infarction was distinctly visible by examining TTC-stained sections, and the pale region represented the focal infarcts.

### 2.7. Neuron Culture and Live-Cell Microscopy

Primary hippocampal neurons were disassociated from embryonic day 18 (E18), plated onto poly-D-lysine (Sigma-Aldrich, USA) coated coverglasses, and cultured in neurobasal medium supplemented with 2% B27 and 1% Glutamax (Life Technology, USA). At 11–14 days* in vitro* (DIV), cells were transfected with adenovirus expressing superecliptic pHluorin (SEP) tagged GluN1 and 48 hr later with adenovirus expressing GluN2A. The sequences encoding GluN1 and GluN2A were obtained from the plasmid pCI-SEP-NR1 (plasmid 23999, Addgene, USA) and pCI-SEP-NR2A (plasmid 23997, Addgene) contributed by Kopec et al. [23].

A Ti-E inverted fluorescence microscope with a Perfect Focus System (Nikon, Japan) was employed. Images were collected through a 100x oil-immersion objective (Plan Apo, NA. = 1.45, Nikon) and recorded by a cooled CCD (Orca-ER, Hamamatsu, Japan). 48 hr after GluN2A transfection (DIV15-18), coverglasses with hippocampal neurons were placed in an imaging chamber (AC-PI, Live Cell Instrument, South Korea) and perfused with the extracellular solution (ECS) containing (in mM) 140 NaCl, 5 KCl, 1.3 CaCl_2_, 25 HEPES, 33 glucose, and 1 MgCl_2_ (pH 7.4) at 37°C. During OGD treatment, neurons were perfused with the medium containing sucrose instead of glucose, which had been saturated with 95% N_2_/5% CO_2_. Cells were incubated with Tat-GluN2B for 15 min or not. Subsequently, live-cell imaging was performed on 5 min before and 10 min after ECS (as control) or OGD treatment. The images were processed and analyzed by NIS-element AR software (Nikon) or Fiji software (National Institutes of Health, USA).

### 2.8. Data Analysis

All population data were expressed as mean ± SEM. Paired-Samples *t*-test was used to assess statistical significance and Independent-Samples *t*-test and analysis of variance (ANOVA) were performed to compare between multiple groups. *P* < 0.05 values were accounted for statistical significance, and the significance for homogeneity of variance test was set at 0.1.

## 3. Results

### 3.1. Synaptic Plasticity in NMDAR-Mediated Responses Depends on Duration of OGD and PT Treatment in Hippocampal Slices

**Figure 1:**
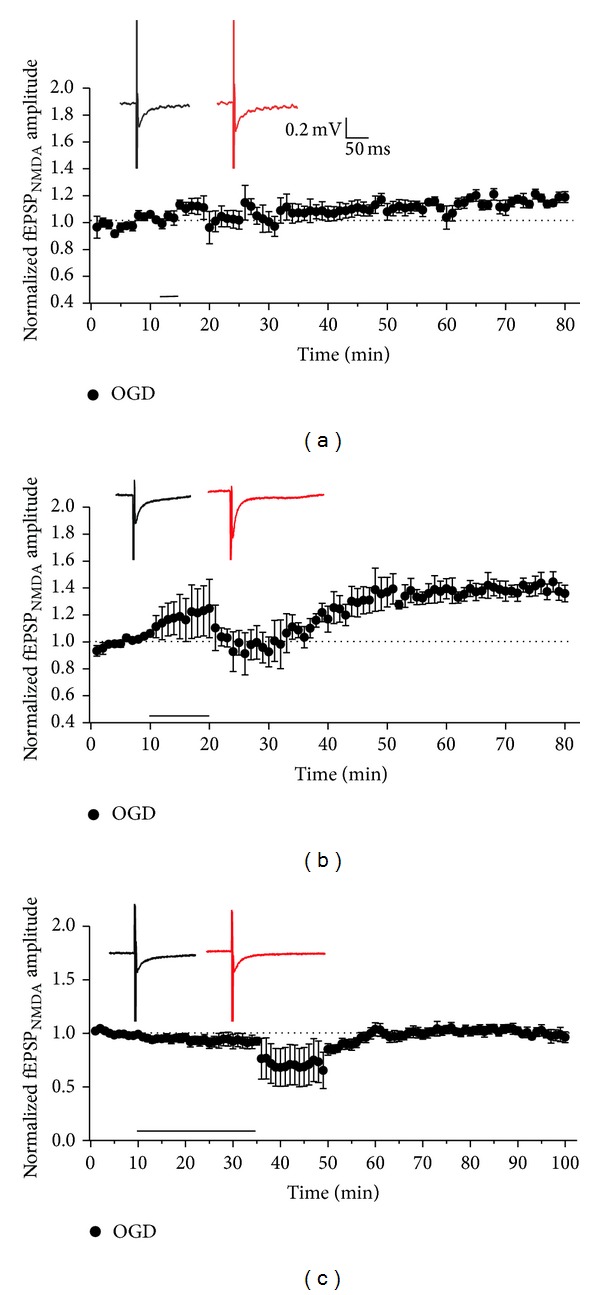
NMDAR-mediated plasticity recorded by fEPSPs is induced using OGD treatment with different time periods. Exposure to 3 min (a), 10 min (b), and 25 min (c) of OGD showed a slight but persistent increase of fEPSP amplitude (1.18 ± 0.03, compared with baseline, *n* = 6, *P* < 0.05), significant potentiation of fEPSP amplitude (1.37 ± 0.07, compared with baseline, *n* = 5, *P* < 0.05), and no obvious effects (1.03 ± 0.04, *n* = 5, *P* > 0.05) on fEPSP amplitude, respectively. Inset: sample traces were from average of the first 10 sweeps in baseline and average of the last 10 sweeps of the whole recordings.

To examine whether plasticity in NMDAR-mediated synaptic responses was affected by duration of OGD treatment, we recorded i-LTP in NMDAR-mediated fEPSPs (NMDA fEPSPs) in acute hippocampal slices [24–27]. NMDA fEPSPs were isolated in the presence of GABA_A_ antagonist BMI (10 *μ*M) and AMPAR antagonist NBQX (10 *μ*M) in low-magnesium ACSF. We found that OGD treatment for different time periods indeed exerted differential effects on inducing NMDAR-mediated i-LTP (NMDA i-LTP). OGD treatment for 3 min only elicited a slight but persistent increase of fEPSP amplitude (Figure 1(a), 1.18 ± 0.03, *n* = 6, *P* < 0.05). When the OGD duration went up to 10 min, a significant potentiation of fEPSP amplitude was observed, which kept stable at least for 30 min in our recordings (Figure 1(b), 1.37 ± 0.07, *n* = 5, *P* < 0.05). When OGD duration went up to 25 min, however, no potentiation in NMDA fEPSPs was detected (Figure 1(c), 1.03 ± 0.04, *n* = 5, *P* > 0.05). These results indicate that the magnitude of NMDA i-LTP differs with OGD duration; 10 min OGD treatment tends to elicit NMDA i-LTP more easily. Shorter or longer OGD treatment seems relatively inefficient to induce NMDA i-LTP.

**Figure 2:**
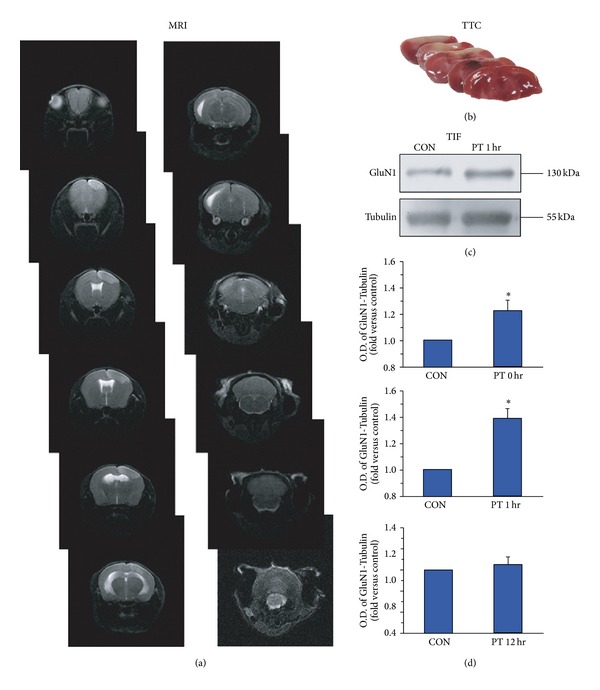
(a) Sequential brain T2-w MRI in an individual mouse with photothrombotic lesion. White arrows indicate infarct region. (b) A series of representative brains stained with TTC after photothrombotic lesion. Pale staining indicates infarct region. (c) Detected by Western blotting, a potentiation of NMDAR in TIF caused by the photothrombotic lesion 1 hr after modeling was shown. (d) Statistical plots of data showing the effects of photothrombotic lesion (0 hr after PT, 1.22 ± 0.08, *n* = 5, *P < 0.05; 1 hr after PT, 1.39 ± 0.07, *n* = 6, *P < 0.05; 12 hr after PT, 1.05 ± 0.07, *n* = 5, *P* > 0.05).

Then we established ischemia model with photothrombotic lesion. We employed sequential brain T2-w MRI and TTC staining method to confirm the infarct region after ischemia modeling (Figure 2(b)). We also employed Western blotting to analyze possible alterations in postsynaptic NMDAR expression. Triton X-100-insoluble fraction (TIF) was used to roughly represent the postsynaptic fraction [21]. Different exposure times were selected, and here 1 hr after PT was showed (Figure 2(c), 0 hr after PT, 1.22 ± 0.08, *n* = 5, *P < 0.05; 1 hr after PT, 1.39 ± 0.07, *n* = 6, *P < 0.05; 12 hr after PT, 1.05 ± 0.07, *n* = 5, *P* > 0.05). A dramatic elevation of postsynaptic expression of GluN1, the obligatory component of NMDAR, was found 1 hr after PT. These data suggest that increased NMDAR number may contribute to NMDA i-LTP.

### 3.2. CaMKII Activity and Autophosphorylation Changed Accompanying NMDAR-Mediated i-LTP in Ischemia

**Figure 3:**
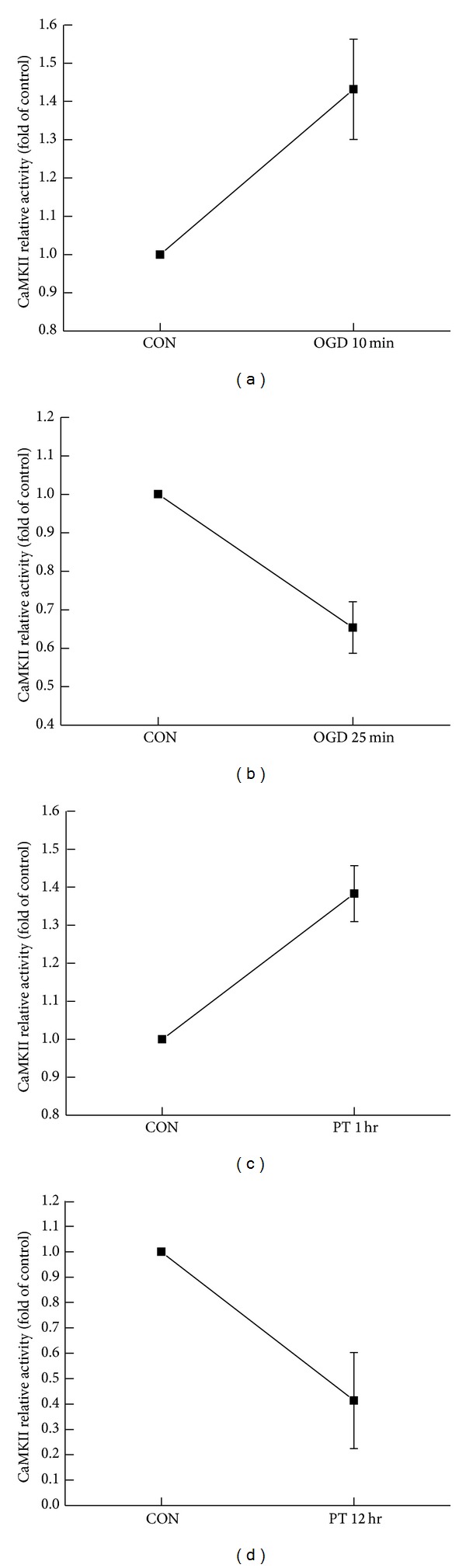
(a) OGD for 10 min caused a significant increase in CaMKII activity (1.43 ± 0.13, *n* = 4, *P* < 0.05). (b) OGD treatment for 25 min led to inhibition of CaMKII activity (0.65 ± 0.07, *n* = 3, *P* < 0.05). (c) 1 hr after PT, CaMKII activity displayed an upward trend (1.38 ± 0.07, *n* = 3, *P* < 0.05). (d) 12 hr after PT, CaMKII activity was inhibited (0.41 ± 0.19, *n* = 3, *P* < 0.05).

**Figure 4:**
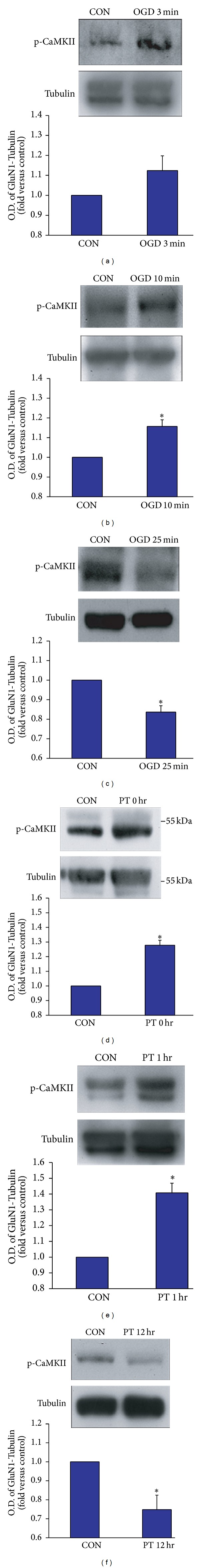
((a)–(c)) The influence of OGD treatment on autophosphorylation of CaMKII (3 min OGD, 1.12 ± 0.07, *n* = 5, *P* > 0.05; 10 min OGD, 1.16 ± 0.03, *n* = 3, *P < 0.05; 25 min OGD, 0.84 ± 0.03, *n* = 5, *P < 0.05). ((d)–(f)) The influence of OGD treatment on autophosphorylation of CaMKII (0 hr after PT, 1.28 ± 0.03, *n* = 4, *P < 0.05; 1 hr after PT, 1.41 ± 0.06, *n* = 5, *P < 0.05; 12 hr after PT, 0.75 ± 0.08, *n* = 5, *P < 0.05).

NMDAR activation and Ca^2+^ influx increase intracellular calcium level. This increased Ca^2+^ binds with calmodulin to form a calcium/calmodulin complex, which in turn activates *α*CaMKII and promotes *α*CaMKII autophosphorylation at T286 site. Increased CaMKII activity and CaMKII autophosphorylation have been reported in OGD and middle cerebral artery occlusion (MCAO) in brain tissue. Here, we examined CaMKII activity in OGD model at different time points following OGD treatment by using Cyclex CaMKII assay kit (following the instructions) to test whether the alteration in CaMKII activity was correlated with duration of OGD treatment. We found that exposure to OGD for 10 min caused a significant increase in CaMKII activity (Figure 3(a), 1.43 ± 0.13, *n* = 4, *P* < 0.05). In contrast, OGD treatment for 25 min led to inhibition of CaMKII activity (Figure 3(b), 0.65 ± 0.07, *n* = 3, *P* < 0.05). In addition, we also test CaMKII activity change at different time points in PT ischemia model. One hour after PT, CaMKII activity displayed an upward trend (Figure 3(c), 1.38 ± 0.07, *n* = 3, *P* < 0.05). In contrast, CaMKII activity decreased markedly 12 hr after PT (Figure 3(d), 0.41 ± 0.19, *n* = 3, *P* < 0.05). Accordingly, we observed an increase in autophosphorylation of CaMKII following either 3 min or 10 min OGD treatment, but detected a decrease in CaMKII autophosphorylation 25 min after OGD treatment, determined by Western blotting assay (Figures 4(a)–4(c), 3 min OGD, 1.12 ± 0.07, *n* = 5, *P* > 0.05; 10 min OGD, 1.16 ± 0.03, *n* = 3, *P < 0.05; 25 min OGD, 0.84 ± 0.03, *n* = 5, *P < 0.05). Similar bidirectional changes in CaMKII autophosphorylation were also observed in PT ischemia model. An increase in CaMKII autophosphorylation was observed immediately (0 hr) or 1 hr after PT, while reduction in CaMKII autophosphorylation was detected 12 hr after PT (Figures 4(d)-4(e), 0 hr after PT, 1.28 ± 0.03, *n* = 4, *P < 0.05; 1 hr after PT, 1.41 ± 0.06, *n* = 5, *P < 0.05; 12 hr after PT, 0.75 ± 0.08, *n* = 5, *P < 0.05).

### 3.3. Active CaMKII Targeting at NMDA Was Involved in Inducing NMDAR-Mediated i-LTP

**Figure 5:**
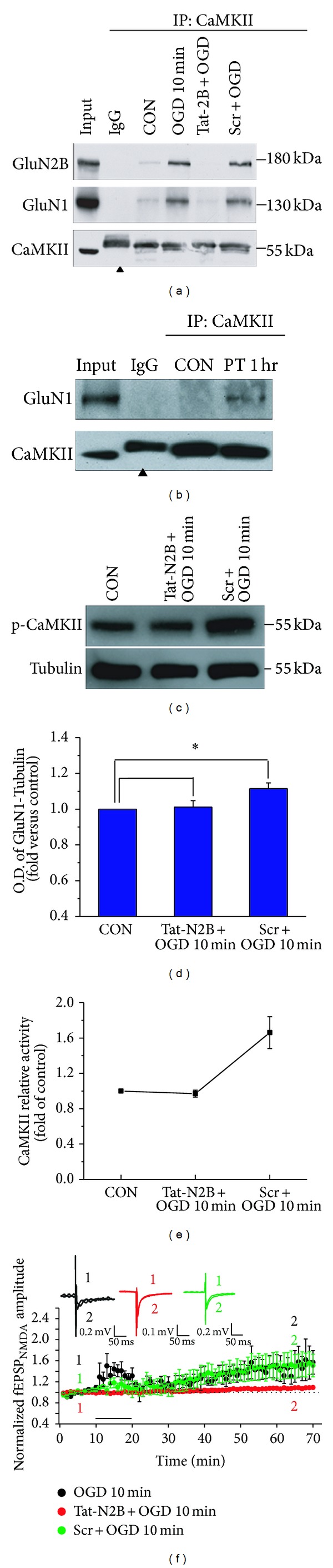
(a) Co-IP assay with anti-CaMKII antibody revealed the association of CaMKII and NMDAR. Peptides occluded the increase of CaMKII and NDMAR by OGD treatment while scramble peptides showed no effect. Arrowhead indicates the nonimmune IgG heavy chain. (b) One hour after photothrombosis caused an increase in the interaction between CaMKII and NMDAR. Arrowhead indicates the nonimmune IgG heavy chain. ((c)-(d)) Peptides Tat-GluN2B suppressed the autophosphorylation of CaMKII in OGD model, compared with control, 1.01 ± 0.04, *n* = 4, *P* > 0.05, while scramble peptides failed to exert any effect on OGD model, compared with control, 1.11 ± 0.03, *n* = 4, *P < 0.05. (e) Peptides Tat-GluN2B decreased the CaMKII relative activity when 10 min OGD was applied, compared with control, 0.97 ± 0.04, *n* = 4, *P* > 0.05, while scramble peptides failed to exert any effect when 10 min OGD was used, compared with control, 1.66 ± 0.18, *n* = 4, *P < 0.05. (f) Peptides Tat-GluN2B led to impaired i-LTP when the hippocampal slice was exposed to OGD for 10 min, *n* = 5, compared with OGD, *n* = 3, 0.73 ± 0.02, *P* < 0.05. Scramble peptides were used when the hippocampal slice was exposed to OGD for 10 min, *n* = 5, compared with OGD, 1.02 ± 0.03, *P* > 0.05. As a control, OGD treatment for 10 min exhibited normal i-LTP. Overlaid traces above the graph showed changes in amplitude of fEPSPs chosen at the times indicated on the graph.

CaMKII activation and subsequent binding of CaMKII with GluN2B subunit of NMDARs are critical to LTP. We employed coimmunoprecipitation (Co-IP) assay to determine the association between CaMKII and NMDAR at the time points in which we observed significant changes in NMDA fEPSPs and CaMKII phosphorylation in OGD or PT ischemia models. As we expected, an increase in association between CaMKII and NMDAR was observed after 10 min of OGD treatment in OGD model or after 1 hr PT in PT model (Figures 5(a)-5(b)).

To further elucidate whether formation of CaMKII and NMDAR complex is required for the induction of pathological plasticity, we utilized short cell-permeable peptides Tat-GluN2B derived from GluN2B binding sequence (1295–1309) with CaMKII to specially interfere the association between CaMKII and NMDARs. Tat protein (Tyr-Gly-Arg-Lys-Lys-Arg-Arg-Gln-Arg-Arg-Arg), which was obtained originally from the cell-membrane transduction domain of the human immunodeficiency virus-type 1 (HIV-1), was fused to the constructed peptides and resulted in fusion of Tat-GluN2B peptides. This manipulation allowed the constructed peptides to easily cross the membrane and exert their effects intracellularly [28]. As expected, disturbing the CaMKII-NMDAR interaction with the Tat-GluN2B peptide decreased the association between CaMKII and NMDAR, while scramble peptides failed to display any effect on the association (Figure 5(a)). Interestingly, Tat-GluN2B application also decreased the CaMKII autophosphorylation to baseline level, while scramble peptides failed to exert any effect (Figures 5(c)-5(d), 1.01 ± 0.04, *n* = 4, *P* > 0.05; 1.11 ± 0.03, *n* = 4, *P < 0.05), and the CaMKII activity was reduced to the level of control group when Tat-GluN2B was applied, but scramble peptides failed to exert any effect (Figure 5(e), 0.97 ± 0.04, *n* = 4, *P* > 0.05; 1.66 ± 0.18, *n* = 4, *P < 0.05). It was observed that there was a decrease in the amplitude of NMDAR-mediated i-LTP after disturbing the connection between CaMKII and NMDAR with peptides Tat-GluN2B (Figure 5(f), *n* = 5, compared with OGD, 0.73 ± 0.02, *P* < 0.05). But scramble peptides failed to exert any effect when the hippocampal slice was exposed to OGD for 10 min (Figure 5(f), *n* = 5, compared with OGD, 1.02 ± 0.03, *P* > 0.05). In line with the changes in CaMKII activity and autophosphorylation, we found that NMDA i-LTP induced by 10 min OGD treatment was also reversed by Tat-GluN2B incubation throughout the recording.

**Figure 6:**
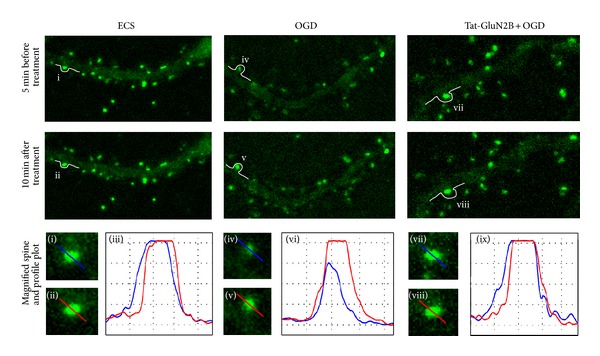
The expression of NMDAR at spine was induced by OGD 2 min treatment and suppressed by Tat-GluN2B incubation. SEP-GluN1 transfected neurons were incubated with Tat-GluN2B for 15 min or not. Subsequently, imaging was performed on 5 min before (first row) and 10 min after (second row) ECS (as control) or OGD 2 min treatment. Magnified images were corresponding to the spines denoted by lowercase Roman numerals ((i), (ii), (iv), (v), (vii), and (viii)). Profile plots were corresponding to the blue and red lines in their left two magnified images ((iii), (vi), and (ix)).

We next examined whether OGD-induced NMDA i-LTP was caused by NMDAR insertion into postsynaptic membrane. We transfected cultured hippocampal cells with GluN1 that is tagged by a pH-sensitive fluorescent protein SEP and employed live-cell imaging to monitor possible changes in fluorescent intensity on surface of spines, where usually excitatory glutamate synapses are located. As shown in Figure 6, the fluorescent intensity at spines increased 10 min after OGD treatment for 2 min, indicating transportation of GluN1 from intracellular vesicles to the plasma membrane of spine. This GluN1 trafficking and subsequent increase in postsynaptic NMDAR number may underlie NMDAR-mediated i-LTP. In addition, disturbing the association between CaMKII and GluN2B with Tat-GluN2B reversed the increase in SEP-GluN1 fluorescent intensity. These results suggest that OGD treatment induced new NMDAR trafficking to spine membrane, and CaMKII-GluN2B association is required for this NMDAR trafficking.

## 4. Discussion

In this study, we used OGD modeling* in vitro* ischemia on the mouse hippocampal slices and photothrombotic lesion modeling* in vivo* ischemia in the mouse hippocampus. As we know, OGD had effects on brain regions including hippocampus to induce NMDAR-mediated i-LTP. But in mounting studies, PT was usually used to research cortical lesion [29, 30]. Therefore it is critical to determine whether hippocampus really suffered from ischemia induced by PT in our experiment. To solve this problem, focal illumination was set to the maximum to widen and deepen the volume of injury to make sure hippocampus suffered from this lesion, but the intensity was set within the extent mice could endure. To examine the lesions of PT, T2-w MRI and TTC staining were conducted after the treatment and the infract region was directly viewed from the photographs.

This study exhibited that NMDAR-mediated i-LTP depends on duration of OGD and PT treatment. Exposure to OGD for 3 min induced a mild increase in i-LTP, while the activity and autophosphorylation level of CaMKII did not display the same trend likewise. Through analysis, it may include two possibilities: one is that the CaMKII assay kit and Western blotting were less sensitive than electrophysiology; the other one is, together with active CaMKII, other mechanisms participated in the NMDAR-mediated i-LTP in 3 min exposure to ischemia.

Multiple evidences reported that the downstream reaction triggered by active CaMKII was involved in synaptic plasticity, LTP, and LTD. And CaMKII was observed to be activated in ischemia exposure. It was consistent with our experiment that targeting of active CaMKII to NMDAR has been proved to be important in i-LTP. Tat-GluN2B was to disturb the interaction of CaMKII and NMDAR, and this result has been shown in coimmunoprecipitation. Moreover, 10 min OGD duration with peptides Tat-GluN2B irrigation exhibited no increase in the amplitude of fEPSPs; we can concluded that the Tat-GluN2B puts its effects on CaMKII activity, autophosphorylation levels, and NMDAR-mediated i-LTP. But the decreased activity of CaMKII by Tat-GluN2B is always lower than the untreated one. There is connection between CaMKII and NMDAR in normal situation, while the interaction may be more compact or the quantity may be even more after the hippocampus was injured. Before exposure to OGD, Tat-GluN2B was preincubated and then may block the new forming of the complex as well as block the original complex; both cooperate to cause the suppressed activity of CaMKII.

In conclusion, it was determined that NMDAR-mediated i-LTP was induced by different durations of OGD and PT. Meanwhile, CaMKII was activated and autophosphorylated. And our data found the interaction between CaMKII and NMDARs to be promoted by active CaMKII, which was disturbed by Tat-GluN2B after OGD. A similar mechanism was suggested in PT modeling. In earlier studies, as a traditional induction method, OGD was applied to research on hippocampal ischemia widely, but few researchers integrate the corporate results in OGD and PT, a modeling* in vivo*. Hence, our results will provide a novel sight into ischemic research.

## References

[1] Li L, Qu W, Zhou L (2013). Activation of transient receptor potential vanilloid 4 increases nmda-activated current in hippocampal pyramidal neurons. *Frontiers in Cellular Neuroscience*.

[2] Calabresi P, Centonze D, Pisani A, Cupini LM, Bernardi G (2003). Synaptic plasticity in the ischaemic brain. *The Lancet Neurology*.

[3] Hsu K-S, Huang C-C (1997). Characterization of the anoxia-induced long-term synaptic potentiation in area CA1 of the rat hippocampus. *British Journal of Pharmacology*.

[4] Mattson MP, Cheng B, Culwell AR, Esch FS, Lieberburg I, Rydel RE (1993). Evidence for excitoprotective and intraneuronal calcium-regulating roles for secreted forms of the *β*-amyloid precursor protein. *Neuron*.

[5] Bayer KU, LeBel É, McDonald GL, O’Leary H, Schulman H, De Koninck P (2006). Transition from reversible to persistent binding of CaMKII to postsynaptic sites and NR2B. *Journal of Neuroscience*.

[6] Dixon RM, Mellor JR, Hanley JG (2009). PICK1-mediated glutamate receptor subunit 2 (GluR2) trafficking contributes to cell death in oxygen/glucose-deprivedhippocampal neurons. *Journal of Biological Chemistry*.

[7] Crepel V, Hammond C, Chinestra P, Diabira D, Ben-Ari Y (1993). A selective LTP of NMDA receptor-mediated currents induced by anoxia in CA1 hippocampal neurons. *Journal of Neurophysiology*.

[8] Crepel V, Hammond C, Krnjevic K, Chinestra P, Ben-Ari Y (1993). Anoxia-induced LTP of isolated NMDA receptor-mediated synaptic responses. *Journal of Neurophysiology*.

[9] Szydlowska K, Tymianski M (2010). Calcium, ischemia and excitotoxicity. *Cell Calcium*.

[10] Jourdain P, Fukunaga K, Muller D (2003). Calcium/calmodulin-dependent protein kinase II contributes to activity-dependent filopodia growth and spine formation. *Journal of Neuroscience*.

[11] Picconi B, Tortiglione A, Barone I (2006). NR2B subunit exerts a critical role in postischemic synaptic plasticity. *Stroke*.

[12] Yao W, Ji F, Chen Z (2012). Glycine exerts dual roles in ischemic injury through distinct mechanisms. *Stroke*.

[13] Barria A, Malinow R (2005). NMDA receptor subunit composition controls synaptic plasticity by regulating binding to CaMKII. *Neuron*.

[14] Meng F, Guo J, Zhang Q, Song B, Zhang G (2003). Autophosphorylated calcium/calmodulin-dependent protein kinase II*α* (CaMKII*α*) reversibly targets to and phosphorylates N-methyl-D-aspartate receptor subunit 2B (NR2B) in cerebral ischemia and reperfusion in hippocampus of rats. *Brain Research*.

[15] Meng F, Zhang G (2002). Autophosphorylated calcium/calmodulin-dependent protein kinase II *α* induced by cerebral ischemia immediately targets and phosphorylates N-methyl-D-aspartate receptor subunit 2B (NR2B) in hippocampus of rats. *Neuroscience Letters*.

[16] Colbran RJ, Brown AM (2004). Calcium/calmodulin-dependent protein kinase II and synaptic plasticity. *Current Opinion in Neurobiology*.

[17] Leonard AS, Bayer KU, Merrill MA (2002). Regulation of calcium/calmodulin-dependent protein kinase II docking to N-methyl-D-aspartate receptors by calcium/calmodulin and *α*-actinin. *Journal of Biological Chemistry*.

[18] Strack S, Colbran RJ (1998). Autophosphorylation-dependent targeting of calcium/calmodulin-dependent protein kinase II by the NR2B subunit of the N-methyl-D-aspartate receptor. *Journal of Biological Chemistry*.

[19] Nisticò R, Piccirilli S, Cucchiaroni ML (2008). Neuroprotective effect of hydrogen peroxide on an in vitro model of brain ischaemia. *British Journal of Pharmacology*.

[20] Skelding KA, Spratt NJ, Fluechter L (2012). Alphacamkii is differentially regulated in brain regions that exhibit differing sensitivities to ischemia and excitotoxicity. *Journal of Cerebral Blood Flow and Metabolism*.

[21] Yan J-Z, Xu Z, Ren S-Q (2011). Protein kinase C promotes N-methyl-D-aspartate (NMDA) receptor trafficking by indirectly triggering calcium/calmodulin-dependent protein kinase II (CaMKII) autophosphorylation. *Journal of Biological Chemistry*.

[22] Hédou GF, Koshibu K, Farinelli M (2008). Protein phosphatase 1-dependent bidirectional synaptic plasticity controls ischemic recovery in the adult brain. *Journal of Neuroscience*.

[23] Kopec CD, Li B, Wei W, Boehm J, Malinow R (2006). Glutamate receptor exocytosis and spine enlargement during chemically induced long-term potentiation. *Journal of Neuroscience*.

[24] Frantseva MV, Carlen PL, El-Beheiry H (1999). A submersion method to induce hypoxic damage in organotypic hippocampal cultures. *Journal of Neuroscience Methods*.

[25] Pugliese AM, Coppi E, Spalluto G, Corradetti R, Pedata F (2006). A3 adenosine receptor antagonists delay irreversible synaptic failure caused by oxygen and glucose deprivation in the rat CA1 hippocampus in vitro. *British Journal of Pharmacology*.

[26] Sun X, Yao H, Douglas RM, Gu XQ, Wang J, Haddad GG (2010). InsulinPI3K signaling protects dentate neurons from oxygen-glucose deprivation in organotypic slice cultures. *Journal of Neurochemistry*.

[27] Yin HZ, Sensi SL, Ogoshi F, Weiss JH (2002). Blockade of Ca2+-permeable AMPA/kainate channels decreases oxygen-glucose deprivation-induced Zn2+ accumulation and neuronal loss in hippocampal pyramidal neurons. *Journal of Neuroscience*.

[28] Hashida H, Miyamoto M, Cho Y (2004). Fusion of HIV-1 Tat protein transduction domain to poly-lysine as a new DNA delivery tool. *British Journal of Cancer*.

[29] Lee J-K, Kim J-E, Sivula M, Strittmatter SM (2004). Nogo receptor antagonism promotes stroke recovery by enhancing axonal plasticity. *Journal of Neuroscience*.

[30] Lipsanen A, Flunkert S, Kuptsova K (2013). Non-selective calcium channel blocker bepridil decreases secondary pathology in mice after photothrombotic cortical lesion. *PLoS ONE*.

